# Predicting dry matter intake in gestating ewes using greenhouse gas measurements from portable accumulation chambers

**DOI:** 10.1093/jas/skag158

**Published:** 2026-05-16

**Authors:** Gareth F Difford, Jette Jakobsen, Ingjerd Dønnem, Geir Steinheim, Bente A Åby

**Affiliations:** Institute of Animal and Aquacultural Sciences, Norwegian University of Life Sciences, Ås, N-1432, Norway; The Norwegian Association of Sheep and Goat Breeders, Ås, N-1430, Norway; Institute of Animal and Aquacultural Sciences, Norwegian University of Life Sciences, Ås, N-1432, Norway; Institute of Animal and Aquacultural Sciences, Norwegian University of Life Sciences, Ås, N-1432, Norway; Institute of Animal and Aquacultural Sciences, Norwegian University of Life Sciences, Ås, N-1432, Norway

**Keywords:** carbon dioxide, dry matter intake, methane, machine learning, portable accumulation chambers, sheep

## Abstract

Accurate measurement of dry matter intake (DMI) in sheep is logistically challenging and costly, particularly under commercial conditions and across diverse production systems. This study evaluated whether methane (CH_4_), carbon dioxide (CO_2_) and oxygen (O_2_) gas measurements from portable accumulation chambers (PAC), combined with bodyweight (BW) and eating time (ET), can predict DMI in gestating ewes. Two indoor feeding trials were conducted at a single research facility using Norwegian White Sheep (NWS) and Old Norwegian Spaelsau (ONS). Trial 1 used two grass silage qualities in a 2 × 2 factorial design; Trial 2 used fresh-cut herbage in a repeated-measures design. Ewes (*n* = 40 per trial; 29 in common) were individually housed with access to one feed bin and fed *ad libitum*. Daily DMI was recorded using automated feed bins, and PAC measurements of CH_4_, CO_2_, and O_2_ were collected on 20 d per ewe per trial. After quality control, 1,830 ewe d records remained. Supervised learning models were trained on ewe-identity–blocked training sets and evaluated on external test sets stratified by trial and breed. In this particular dataset, random forest (RF) models outperformed regularized regression, partial least squares, k-nearest neighbors, support vector machines, and a deep neural network. The best RF model, using PAC traits, LW, and ET (Pred_DMI3), achieved R^2^ = 0.77, root mean square error of 279 g/d, and mean absolute percentage error = 13.8% in the combined test set. Models using only PAC traits performed comparably to models including BW and ET with only marginal losses in validation metrics. Mixed-model analyses indicated strong individual-level correlations between predicted and observed DMI (rᵢ = 0.79–0.81) after adjusting for breed, diet, and trial. These results demonstrate that short-term PAC-derived gas traits can provide scalable proxies for daily DMI in gestating ewes under controlled indoor conditions, with potential application in intake phenotyping and benchmarking.

## Teaser

We show that simple gas measurements from portable chambers can be used to predict how much feed gestating ewes eat each day, opening a practical route to indicate feed intake and feed efficiency.

## Introduction

Accurate measurement of dry matter intake (DMI) is central to understanding feed efficiency, animal metabolism, and environmental impact in ruminants. In practice, however, direct measurement of individual DMI in sheep is logistically difficult and prohibitively expensive at scale, especially in extensive, roughage, and pasture-based systems like those in Norway (Robinson et al 2016). Automated feed bins with radio-frequency identification (RFID) head gates have increased the feasibility of intake recording (Pinares-Patiño et al 2013; Johnson et al 2022), but their deployment at scale is still limited. This has motivated growing interest in biologically meaningful proxy traits, including body weight (BW) dynamics, feeding behavior, and short-term gaseous exchange measured in portable accumulation chambers (PAC).

Short-term measurements from PAC provide one such proxy by quantifying methane (CH_4_), carbon dioxide (CO_2_), and oxygen (O_2_) exchange. These gaseous traits are closely related to feed intake rate, metabolic heat production, and energy turnover. In addition, PAC-derived CH_4_ and CO_2_ emissions show moderate heritability (0.20 ± 0.05) and positive genetic correlations (0.67 ± 0.11) with respiration-chamber measurements (Jonker et al 2018). These relationships have been confirmed across sheep life stages in pasture conditions (Connor et al 2024), supporting the use of PAC emissions as scalable, biologically meaningful proxies for intake and efficiency phenotyping. Feeding behaviors such as time spent eating have been any area of research as proxies for feed intake, feed efficiency, and methane emissions (Cammack et al 2005; Etancelin et al 2019; Sepulveda et al 2022) and were selected, as these can be recorded from indoor automatic feeding bins as well as halter-mounted sensors-based technologies in the field (Versluijs et al 2023).

Norwegian sheep production is highly seasonal and regionally variable, with flocks alternating between indoor feeding on conserved forages and summer grazing on cultivated infields and extensive mountain outfields (Holmøy and Waage 2015). Unlike many major sheep-producing countries where animals remain under closer managerial control year-round, Norwegian sheep often graze freely on primarily unfenced remote mountain uncultivated pastures during summer, with limited opportunity to monitor diet selection or intake. These contrasting and largely unmanaged grazing conditions make direct quantification of DMI particularly challenging and increase the value of indirect estimation methods.

Previous studies show that the association between DMI, BW, and gaseous emission depends on breed, age, and feeding system (Robinson et al 2020). High correlations between CO_2_ and DMI have been reported in Norwegian and international settings (Graverand et al 2025). Traits like CH_4_ emissions and eating time (ET), are correlated to DMI, this relationship is influenced by dietary composition, such as neutral detergent fiber concentration (Huhtanen et al 2019; Sepulveda et al 2022). It is only possible to obtain concurrent repeated records of these traits when pregnant ewes are housed indoors during the winter and train prediction models for DMI. Very little is known about model prediction performance for DMI under these conditions. Potentially with additional efforts and the flexibility afforded by PACs and sensor-based technologies such as virtual fencing collars, it may be possible to obtain scalable prediction of DMI under more extensive conditions.

To promote generalizability of model prediction performance, the training data should span the biological and environmental diversity under which predictions will be applied. At the same time, validation must preserve independence between training and testing observations to avoid inflated estimates of predictive performance. The Norwegian White Sheep (NWS) and Old Norwegian Spael (ONS) provide a useful contrast for evaluating these relationships. NWS is a modern composite breed selected for growth and carcass traits, whereas ONS is a smaller, unimproved landrace commonly kept under extensive conditions (Åby et al 2023). Since individual feed intake can only be measured when animals are housed indoors and typically fed silage, extending prediction to grazing conditions requires replicating aspects of the grazing diet under controlled conditions by providing freshly harvested grass. However, the environmental variation required to promote generalizability may also inflate phenotypic associations between predictors and DMI, reducing their suitability as proxies for genetic ranking.

When repeated measurements are available per individual, conventional cross-validation can overestimate predictive accuracy if records from the same individual appear in both training and validation sets. We therefore apply novel identity-blocked cross-validation to preserve independence at the individual level. Furthermore, when the goal is to rank animals for genetic selection on a predicted phenotype, careful attention to validation design is required to separate individual-level variation from environmental effects and to ensure predictive models perform reliably within breed (Difford et al 2019; Jonker et al 2021). This requires explicit distinguishing between phenotypic prediction accuracy, which may be influenced by environmental effects, and individual ranking potential, which reflects the consistency of individual differences, and evaluating whether models capture stable individual-level signals relevant for breeding applications.

Therefore, the objective of this study was to evaluate the ability of PAC-derived CH_4_, CO_2_, and O_2_ emissions, together with BW and ET, to predict DMI in gestating NWS and ONS ewes housed indoors and offered either two contrasting silage qualities or fresh-cut grass. We implemented identity-blocked cross-validation to account for the repeated measurements and explicitly assessed both phenotypic prediction accuracy and ranking potential. In addition, we evaluated individual-level correlations between observed and predicted DMI as indicators of suitability for breeding applications. We hypothesized that: 1) PAC emissions alone would predict DMI with moderate to high accuracy, and 2) adding BW and ET would improve prediction and strengthen within-breed ranking potential. The findings aim to inform the development of scalable intake proxies for Norwegian sheep systems and support long-term breeding and climate mitigation strategies.

## Materials and methods

### Experimental design and animal management

The study was conducted in strict compliance with the regulations for experiments on live animals in Norway (FOR-2015-06-18-761) and the EU (Directive 2010/637EU). All animal experimental procedures were approved by the Norwegian Food and Safety Authority for the protection of animals used for scientific purposes (FOTS ID 24196).

Two feeding trials were conducted at the Livestock Production Research Center (SHF), (60°N, 11°E), the Norwegian University of Life Sciences (NMBU), Norway. Both trials had forty ewes from two breeds, twenty NWS and twenty ONS. In the first trial (Trial 1) two grass silage qualities were fed in a two-by-two crossover design (Åby et al 2023). In the second trial (Trial 2) the 40 ewes were allocated to two groups and balanced for breed and offered fresh-cut grass during 6 wk in a repeated measures design (Flesjø 2023). In total 29 ewes were common to both trials.

### Feeding and feed intake measurements

#### Trial 1: Silage diets

Trial 1 is described in detail elsewhere (Åby et al 2023). It utilized two grass silage qualities produced from a field established in 2017, consisting primarily of timothy (*Phleum pratense*) and perennial ryegrass (*Lolium perenne*), with white clover (*Trifolium repens*), dandelion (*Taraxacum officinale*), and northern dock (*Rumex longifolius*). The early-cut silage was harvested during late stem elongation (booting stage; BBCH 41–49), and the late-cut silage was harvested 15 d later at the heading stage (BBCH 55–59). Both were wilted for 24 h to a target of 350 g dry matter/kg, treated with the additive GrasAAT Lacto (73% formic acid, 0.15% lactose), bailed, wrapped in six layers of plastic film, and stored indoors until feeding.

The trial was conducted from November 2020 to January 2021 in two 3-wk periods (one wk of adaptation followed by 2 wk of measurements). Forty ewes in early pregnancy were used in a 2 × 2 crossover factorial design with two breeds (20 NWS; 20, ONS) and two silage qualities. Within each breed, ewes were randomly allocated to one of two treatment sequences so that all animals received both silage qualities across the two periods. Treatment order was balanced within breed. The individual ewe was considered the experimental unit. Animals were housed in individual pens and offered silage *ad libitum* (targeting ∼10% refusals). Feed intake was continuously recorded using the BioControl system (Rakkestad, Norway) with one feed bin per pen. No concentrate was provided.

Weekly silage samples were collected for DM determination by oven-drying subsamples at 103 °C. Daily DMI (g/d) was calculated as feed offered minus feed refused, multiplied by DM concentration. BW was recorded before each PAC session using an RFID-equipped walk-through scale. Eating time (ET, min/d) was derived from RFID time stamps indicating the duration for which the ewe’s head was in the feed bin.

#### Trial 2: Fresh-cut herbage

Trial 2 was conducted from August to September 2020 using daily harvested fresh-cut herbage from a 10.3 ha field established in 2018 with the seed mixture *Spire Surfôr Beite Pluss 10* (Felleskjøpet Agri SA, Oslo, Norway), containing 45% timothy (*cv. Liljeros*), 20% meadow fescue (*cv. Vestar*), 15% smooth meadow-grass (*cv. Knut*), 10% perennial ryegrass (*cv. Figgjo*), and 10% white clover (*cv. Edith*). Dandelion and northern dock occurred naturally. Approximately 400 kg of fresh herbage was harvested each morning using a Serigstad forage harvester, with half stored at 3 °C for evening feeding to minimize evaporative losses.

Forty gestating ewes were housed in individual pens and fed *ad libitum* (targeting ∼10% refusals) twice daily at 07:00 h and 17:00 h. The 6-wk trial included 1 wk of adaptation and 5 wk of measurements. The same animals were monitored throughout the measurement period, and variables were recorded repeatedly within ewe over time. The individual ewe was considered the experimental unit.

Feed intake was recorded continuously using the BioControl system with one feed bin per pen; no concentrate was provided. Feed-out samples were collected Monday–Friday throughout the trial (*n* = 38 mornings) and on selected evenings (*n* = 12). Grass residue samples were collected on August 31, September 10, and September 17. Subsamples were oven-dried at 103 °C to determine dry matter (DM) determination. Daily DMI (g/d) was calculated as feed offered minus feed refused multiplied by DM concentration. Residue DM corrections were not applied. ET was calculated from RFID reader logs, and BW was measured before each PAC recording as described for Trial 1.

### PAC measurements

The gases: O_2_ consumption, CH_4_, and CO_2_ production were measured using 10 PAC as described by Åby et al (2023) and Levrault et al (2023). The custom-designed welded aluminum chambers had a total volume of 1,146 liters with transparent polycarbonate observation windows. Gas concentrations were measured using an RKI Eagle 2 instrument (Union City, USA). The Eagle 2 sensors were zeroed and calibrated daily using certified calibration gases of known concentration. Instrument drift was checked before and after each trial. Total emissions were calculated accounting for accumulated gas concentrations, chamber volume, air temperature, air pressure, and sheep volume estimated from BW. Individual 50 min measurements were converted to g/h by multiplying by a constant of 1.2 and then corrected to standard temperature and pressure (STP; 273.15 K and 101.325 kPa) using ideal gas laws. In trial 1 PAC measurements were recorded daily during two sets of two measurement wk (Monday–Friday) for each diet, resulting in 20 measurements per ewe (800 measurements in total). In trial 2, PAC measurements were recorded daily during every second wk of the 6-wk experimental period (Monday–Friday). As multiple PAC measurements could be obtained per ewe per day, this resulted in 30 measurements per ewe (1,200 measurements in total).

### Data preprocessing and quality control

A total of 1,855 observations were available across the two trials after removing incomplete measurement days (ie the last day in both experiments). Multivariate outliers were identified using Wilks’ method (Wilks 1963), which employs Mahalanobis distance to detect observations that deviate significantly from the multivariate mean. The method computed F-statistics for each observation, with those having *P*-values < 0.001 considered outliers and removed from subsequent analyses. This procedure utilized CH_4_, O_2_, CO_2_, BW, and DMI, resulting in 1,830 observations remaining for model development.

### Data partitioning strategy for prediction and validation

A specialized partitioning strategy was implemented to ensure robust cross-validation while accounting for the longitudinal nature of the data. Training and test sets were constructed based on individual ewe identity to prevent data leakage (repeated measurements from any ewe in both the training and the validation). The dataset was divided into: 1) a training set containing 29 animals present in both trials (73% of total observations) and 2) a test set with 22 ewes only found in one of the two trials, comprising 27% of the total observations (Table 1). Test set 1 was further subdivided into a test set that included all ewes on different silages, a test set with all ewes on fresh-cut grass, and finally, two sets including all NWS or all ONS sheep. This partitioning strategy allowed for robust and stratified evaluation of model performance across feeding regimes and genetic backgrounds, supporting the assessment of model generalizability across contexts. The best-performing prediction model will then be used to evaluate ranking of the predicted phenotypes using a repeated-out-of-sample partitioning strategy, described below.

**Table 1:** Overview of ewe and record counts (records in parentheses) across two feeding trials (Trial 1: silage; Trial 2: fresh-cut grass) and two breeds: NWS and ONS.

Dataset subset	Trial 1 silage: NWS	Trial 1 silage: ONS	Trial 2 grass: NWS	Trial 2 grass: ONS	Percentage of total
**Entire dataset**	20 (349)	20 (358)	20 (560)	20 (560)	100
**Training set**	14 (253)	15 (267)	14 (394)	15 (425)	73
**Test set: all**	6 (96)	5 (91)	6 (166)	5 (135)	27
**Test set: Trial 1**			6 (166)	5 (135)	17
**Test set: Trial 2**	6 (96)	5 (91)			10
**Test set: NWS**	6 (96)		6 (166)		14
**Test set: ONS**		5 (135)		5 (91)	12

The table shows distributions for the full dataset, the training set, and five test sets used in model evaluation. Percentages represent the proportion of total records included in each subset.

### Statistical modeling approaches

All modeling was conducted in R version (4.5.1). A total of seven prediction models were trained on the training set and evaluated across multiple test sets. Model performance was assessed using three metrics calculated for both training and test datasets: Root Mean Square Error (RMSE) to measure prediction accuracy, coefficient of determination (R^2^) to quantify explained variance, and Mean Absolute Percentage Error (MAPE) to assess relative prediction error. Models were evaluated across all test sets to determine generalization performance across different animals, trials, and breeds.

Three predictor sets were evaluated using the incremental contribution of additional information. Models were first trained using only PAC measurements (CO_2_, O_2_ CH_4_), then with the addition of BW, and lastly with the addition of ET.

Regularized regression models including LASSO and ridge regression (α set to 1 or 0, respectively) were implemented using the glmnet package (Friedman et al 2010). The regularization parameter (λ) was selected using 10-fold cross-validation within the training set, and predictions were generated using the value minimizing cross-validation error. Partial Least Squares Regression (PLSR) was performed using the pls package (Mevik and Wehrens 2007), and the optimal number of components was determined through cross-validation in the training set.

Additional machine learning models were implemented using implemented using the mlr package (Bischl et al 2016). K-Nearest Neighbors (KNN) regression included hyperparameter tuning for the number of neighbors (k), evaluated from 1 to 10 using cross-validation within the training set. Random Forest regression was implemented using the randomForest algorithm within the mlr framework. Models were trained using default randomForest parameters, including 500 trees (ntree = 500) and the default number of predictors sampled at each split (mtry = floor(p/3)), where p is the number of predictors in the model.

Tree-based boosting models were also evaluated. Extreme Gradient Boosting (XGBoost) models were trained using the xgboost package with 100 rounds, a learning rate of 0.1, and max depth of 6. Gradient Boosting Machines (GBM) were also trained using the H2O AutoML framework (Fryda et al 2024), which performed model selection and tuning to minimize training RMSE.

The SVM regression was performed with hyperparameter tuning across kernel types (radial and polynomial), cost parameters (0.01–10), and gamma values (0.001–1) using grid search with cross-validation on the training set. Deep learning artificial neural networks were built using the H2O machine learning platform (Fryda et al 2024). A neural network with rectifier activation and dropout regularization was trained with 100 hidden neurons, 2,000 epochs, and 5-fold cross-validation. A transformer-based Tabular Prior-Data Fitted Network (TabPFN) (Hollmann et al 2025) was implemented through Python using the reticulate interface (Kalinowski et al 2025). The pretrained TabPFN regressor was applied via the tabpfn Python package. This model performs Bayesian inference via a pretrained transformer, enabling strong generalization even with limited predictor inputs. Predictions were generated directly from the pretrained model without task-specific gradient-based training.

### Out-of-sample DMI prediction and mixed model evaluation

Training and test sets were constructed based on out-of-sample test data, which used the individual ewe identity to prevent data leakage. In other words, it was not possible for any repeated measurements of any ewe to be both in the training data or the test data, as this would overinflate model performances. Random forests were selected for subsequent analyses because they provided the highest overall R^2^, lowest MAPE, and RMSE when all predictors were included, while also demonstrating strong generalization across all test sets. Variable importance for the random forest models was assessed using permutation-based importance (% increase in mean squared error) from the random forest implementation. In order to evaluate predicted DMI phenotypes for ranking, it is necessary to evaluate the predictions on unseen data on unseen ewes to prevent overfitting or over optimistic results. Thus, we implemented the random forest approach on a repeated out-of-sample prediction strategy based on ensuring individual ewe identity numbers were never in both the training and test set simultaneously. The full dataset was repeatedly (five iterations) partitioned into training (80%) and test (20%) sets by randomly sampling ewe identity numbers, ensuring no overlap between individuals used for model training and those used for testing in each iteration. In each repeat, a random forest regression model was trained and then used to predict DMI for the animals in the test set. After five iterations the DMI of all ewes had been independently predicted for DMI. This was repeated for each of the three prediction variable sets, resulting in Pred_DMI1, Pred_DMI2, and Pred_DMI3.

Univariate animal repeatability models were used to estimate the variance components whilst controlling for fixed effects. All analyses were performed using DMU version 6 (Madsen and Jensen 2014). The model for all traits is as follows:


$$y_{\mathrm{ijk}}=\;\mu +\;{\mathrm{BxT}}_{i}+\;S_{j}+\;e_{\mathrm{ijk}}$$

where y_ijk_ is the trait of interest (DMI, Pred_DMI1, Pred_DMI2, Pred_DMI3, CH_4_, CO_2_, O_2_, BW, ET), μ is the intercept, BxT is the ith effect of breed nested within Trial treatment group (i = 6 levels), S is the random effect of the jth sheep Sj ∼ ND (0, Iσ^2^s), and e is the residual ∼ ND (0, Iσ^2^e)

Repeatability estimates (t) were obtained from the variance components by using the equation:


$$t=\;\frac{\sigma_{S}^{2}}{\left(\sigma_{S}^{2}+\;\sigma_{e}^{2}\right)}$$

Pairwise bivariate animal repeatability models of the same form were run to estimate co-variance components controlling for fixed effects. Individual-level correlations (rI), were computed as the correlation between random sheep effects using the following equation:


$$r_{I}=\;\frac{\sigma_{S1,S2}^{2}}{\sqrt{\left(\sigma_{S1}+\;\sigma_{S2}\right)}}$$

where $r_{I}$ is the individual-level correlation between trait 1 and trait 2; $\sigma_{s1,s2}$ is the covariance between random sheep effects for the two traits; and $\sigma_{s1}^{2}$ and $\sigma_{s2}^{2}$ are the corresponding between-animal variance components for trait 1 and trait 2, respectively. Traits 1 and 2 refer to the pair of response variables fitted jointly in each bivariate model (eg CH_4_ and CO_2_ emissions, or CH_4_ and DMI). The correlation therefore represents the association between animals after accounting for fixed effects and repeated measurements. The standard errors of the individual-level correlations and repeatability estimates were derived using Taylor series approximations. Marginal R^2^ for the variance explained by fixed effects were calculated as per (Nakagawa and Schielzeth 2013).

## Results

Data distributions and relationships across trials and breeds are summarized in Table 1. The two breeds differed in body size, with NWS ewes averaging 87–91 kg and ONS ewes 58–67 kg across trials (Table 2).

**Table 2:** Descriptive statistics for on-farm measurements and PAC gas exchange data in NWS and ONS across two feeding trials (silage and fresh cut grass).

Trait	DMI, g/d		BW, kg		ET, min		CH_4_, g/h		CO_2_, g/h		O_2_, g/h	
	Mean	CV	Mean	CV	Mean	CV	Mean	CV	Mean	CV	Mean	CV
**Trial 1 NWS**	1,978 ± 364	18.4	91.2 ± 6.5	7.1	201 ± 47	23.4	1.7 ± 0.3	17.8	61.1 ± 25.4	25.4	−56.3 ± 10.6	18.9
**Trial 1 ONS**	1,137 ± 259	22.8	60.3 ± 8.6	14.2	212 ± 58	27.4	0.95 ± 0.2	24.3	36.1 ± 6.1	16.8	−35.6 ± 6.7	18.7
**Trial 2 NWS**	2,654 ± 380	14.3	87.2 ± 9.5	10.9	302.3 ± 43.1	14.3	2.1 ± 0.3	16.2	65.5 ± 13.8	21.0	−63.2 ± 9.3	14.6
**Trial 2 ONS**	1,819 ± 333	18.3	57.9 ± 7.9	13.7	300 ± 53.9	18.0	1.4 ± 0.26	19.2	42.0 ± 7.0	16.7	−45.3 ± 7.1	15.6

Values are presented as means ± standard deviations and coefficients of variation (CV%).

In Trial 1, mean DMI was higher in NWS (1,978 ± 364 g/d) than ONS (1,137 ± 259 g/d) (Table 2). DMI increased by 35% in Trial 2, however, breed-level differences persisted. ET was similar between the two breeds within each trial but was consistently lower in Trial 1 (201–212 min/d) compared to Trial 2 (300–302 min/d), likely reflecting differences in feed characteristics or intake behavior (Table 2).

Methane and CO_2_ production showed patterns broadly consistent with BW and DMI. In Trial 1, mean CH_4_ production was 1.7 ± 0.3 g/h for NWS and 0.95 ± 0.2 g/h for ONS. In Trial 2, average emissions were higher for both breeds, with means of 2.1 ± 0.3 g/h and 1.4 ± 0.26 g/h for NWS and ONS, respectively. Similar patterns were observed for CO_2_ production (Table 2).

**Figure 1:**
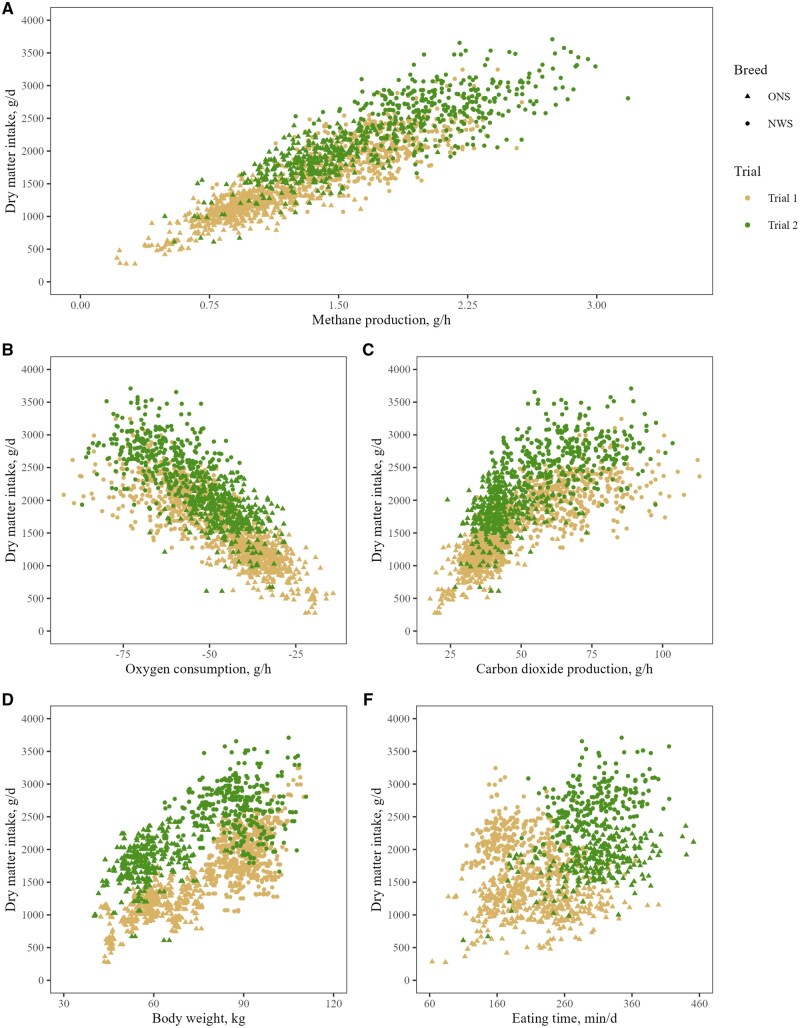
Relationships between dry matter intake, g/d, and A) methane, g/h, B) oxygen consumption, g/h, C) carbon dioxide, g/h, D) body weight, kg, and E) eating time, min/d. Points represent ewe d records for Norwegian White Sheep (NWS, circles) and Old Norwegian Spael (ONS, triangles) in Trial 1 (silage, tan) and Trial 2 (grass, green).

Scatterplots revealed clear relationships between DMI and several predictor traits across trials and breeds (Figure 1). Strong positive associations were observed between DMI and CH_4_ (r = 0.88), CO_2_ (r = 0.71), and BW (r = 0.62). The relationship between DMI and CO_2_ appeared nonlinear, with a plateau at higher intake levels. Oxygen consumption, on the other hand, showed a strong negative trend with DMI (r = −0.80). ET was positive but weakly associated with DMI (r = 0.34), however, distinct clustering by breed and trial was evident (Figure 1).

### Prediction models and validation

The optimal modeling approach varied by predictor configuration: Random forest consistently achieved the highest accuracy when all predictors were available, SVM displayed superior performance when ET was excluded, and TabPFN exhibited the strongest predictive ability in the PAC gas-only scenario (Tables S1–S3). Random forests were selected for subsequent analyses because they provided the highest overall accuracy when all predictors were included, while also demonstrating strong generalization across all test sets. Variable importance analysis indicated that CH_4_ emissions were consistently the most influential PAC-derived predictor across models, followed by O_2_ consumption and ewe BW, with CO_2_ contributing less to model performance. Interestingly, when ET was included in the full model, its importance was comparable to that of CH_4_ (Table S4).

Three random forest models Pred_DMI1, Pred_DMI2, and Pred_DMI3 were developed using progressively richer sets of predictors, and their performance is summarized in Table 3. Each model included the core of PAC traits CH_4_, CO_2_, O_2_, while Pred_DMI2 added BW, and Pred_DMI3 further incorporated ET. As the number of predictors increased, model performance improved consistently, particularly in external test sets.

**Table 3:** Predictive performance of three random forest models for estimating DMI in ewes using different sets of predictor variables.

Prediction model	**Pred_DMI1**			Pred_DMI2			Pred_DMI3		
Predictors	CH_4_, O_2_, CO_2_			CH_4_, O_2_, CO_2_, BW			CH_4_, O_2_, CO_2_, BW, ET		
	*R* ^2^	RMSE	MAPE	*R* ^2^	RMSE	MAPE	*R* ^2^	RMSE	MAPE
**Train**	0.96	126	5.6	0.97	120	5.3	0.98	96	4.2
**Test set: all**	0.73	303	14.3	0.72	307	15.0	0.77	278	13.8
**Test set: Trial 1**	0.65	334	12.5	0.68	321	12.0	0.72	299	11.1
**Test set: Trial 2**	0.61	282	15.5	0.57	298	16.9	0.66	264	15.5
**Test set: NWS**	0.48	352	13.8	0.49	348	13.9	0.60	309	12.0
**Test set: ONS**	0.64	233	14.9	0.58	250	16.4	0.62	237	16.0

Model performance is evaluated using *R*^2^, root mean square error (RMSE, g/d), and mean absolute percentage error (MAPE, %) across the training set and five external test sets.

While all models performed well on the training data (R^2^ between 0.96 and 0.98), indicating strong fit to the observed data, external validation revealed differences in generalizability. Pred_DMI3 achieved the highest performance across the combined test sets, with R^2^ = 0.77, RMSE = 278 g/d, and MAPE = 13.8%. This indicates that the inclusion of on-farm traits such as BW and ET contributes additional information that improves model performance on unseen data.

Test set performance varied when stratified by trial and breed. Across trials, predictions were more accurate in Trial 1 (silage diet) compared to Trial 2 (fresh grass diet). Across breeds, models generally predicted DMI more accurately in ONS ewes than in NWS. For example, in the best-performing model (Pred_DMI3), R^2^ was similar between breeds, with 0.60 for NWS and 0.62 ONS, but the mean absolute percentage error (MAPE) was lower for NWS (12.1%) than for ONS (16.0%), indicating better relative accuracy in predicting DMI in NWS ewes.

### Trait repeatability and correlation with DMI

Linear mixed models indicated that a substantial proportion of the variation in key traits was explained by fixed effects, such as breed and diet within trial (Table 4). Marginal R^2^ values (R^2^LMM) were highest for BW (0.77), followed by DMI (0.75), predicted DMI traits (Pred_DMI1-Pred_DMI3: 0.75–0.78), CH_4_ (0.69), and ET (0.62). Notably, R^2^LMM for predicted DMI traits increased progressively with the addition of predictors, rising from 0.75 in Pred_DMI1 (PAC traits only) to 0.78 in Pred_DMI3 (PAC + BW + ET), indicating the addition of BW and ET improved the explanatory power of the fixed effect portion of variation in DMI.

**Table 4:** Variance components, marginal coefficient of determination ($R_{M}^{2}$), repeatability, and correlations with DMI, for predicted DMI values from random forest models (Pred_DMI1, Pred_DMI2, Pred_DMI3), PAC-derived phenotypes (CH_4_, CO_2_, O_2_), and on-farm traits (BW and ET).

Metrics^1^	$R_{M}^{2}$	Var_ewe_	Var_e_	*t* ± SE	R_P_ with DMI	R_I_ with DMI
**DMI**	0.75	49,278	50,844	0.49 ± 0.05	–	–
**Pred_DMI1**	0.75	39,400	41,117	0.49 ± 0.05	0.61 ± 0.02	0.79 ± 0.06
**Pred_DMI2**	0.76	32,695	37,566	0.47 ± 0.05	0.60 ± 0.02	0.81 ± 0.05
**Pred_DMI3**	0.78	28,773	35,219	0.45 ± 0.05	0.62 ± 0.02	0.79 ± 0.06
**CH_4_**	0.69	0.042	0.035	0.55 ± 0.05	0.62 ± 0.02	0.76 ± 0.07
**CO_2_**	0.60	47.0	67.9	0.41 ± 0.05	0.33 ± 0.02	0.62 ± 0.09
**O_2_**	0.65	31.9	34.9	0.48 ± 0.05	−0.40 ± 0.02	−0.62 ± 0.09
**BW**	0.77	58.1	12.7	0.82 ± 0.03	0.30 ± 0.02	0.41 ± 0.12
**ET**	0.62	770.2	1,069.2	0.42 ± 0.05	0.28 ± 0.02	0.01 ± 0.15

1 $R_{M}^{2}$ = marginal coefficient of determination showing explained variation by fixed effects, Var_ewe_ = individual ewe level variance, Var_e_ = residual variance, *t* ± SE is the repeatability and standard error, R_P_ with DMI is the phenotypic correlation with DMI after correction for fixed effects and R_I_ is the individual level correlation with DMI after correction for fixed effects. Pred_DMI from CH_4_, O_2_, CO_2_; Pred_DMI2 adds BW; Pred_DMI3 further includes ET.

All traits were significantly repeatable after accounting for fixed effects, with repeatability estimates (t ± SE) ranging from 0.41 (CO_2_ ± 0.05) to 0.82 (BW ± 0.03). Similarly, DMI itself showed moderate repeatability (t = 0.49 ± 0.05), while CH_4_ production had the highest repeatability among the PAC traits (t = 0.55 ± 0.05), underscoring its potential as a consistent indicator across individuals.

Phenotypic correlations (R_P_) between measured DMI and all other measured and predicted traits, after correction for fixed effects, ranged from −0.40 (O_2_) to 0.62 (CH_4_ and Pred_DMI3). The predicted DMI traits exhibited the strongest correlations with actual DMI (R_P_ = 0.60–0.62), surpassing those of individual PAC or on-farm traits. Individual-level correlations (R_I_) with measured DMI revealed similar trends but stronger correlations with predicted DMI values (R_I_ = 0.79–0.81) and CH_4_ (R_I_ = 0.76) showed the strongest associations, while ET showed almost no correlation (R_I_ = 0.01 ± 0.15), despite its modest phenotypic association (R_P_ = 0.28).

## Discussion

Our results show that DMI in gestating NWS and ONS ewes can be predicted with reasonable accuracy from phenotypic proxies under silage and freshly cut grass diets. The random forest models achieved R^2^ values of 0.72–0.77 and MAPE of 13.8%–15.1% in the independent test data of this particular dataset and validation design. These levels of performance are comparable to, or slightly better than, those reported in recent multi-country and multi-breed studies on growing sheep, where validation accuracies typically ranged from R^2^ = 0.12–0.37 for models including BW and PAC traits (Graverand et al 2025; Amarilho-Silveira et al 2025) further predicted metabolizable energy intake in lambs and hoggets from 1,708 observations from three breeds (Australian Merino, Corriedale, and Dohne Merino sheep). They achieved a k-fold cross validation R^2^ of 0.91 using SVM and a suite of 15 predictors, which included PAC phenotypes and BW phenotypes on test (Amarilho-Silveira et al 2025). It would be of value to determine if the impressive model performance of Amarilho-Silveira et al (2025) is due to using metabolizable energy intake instead of DMI and thus leveraging a correction for diets into the feed intake phenotype or it is due to a richer predictor set of 15 variables. In contrast, we had 1,830 observations from 51 ewes of two sheep breeds under two contrasting different diets and feeding regimes. Despite the modest number of unique animals, individual-ID-blocked training and testing ensured independence between datasets and prevented data leakage, yielding conservative and realistic estimates of model performance. Daily DMI estimates for the fresh-herbage period were based on feed-out DM and limited residue DM sampling, with DM concentration determined by oven-drying representative samples at 103 °C. As moisture loss and diurnal variation in herbage water content can introduce small errors in as-fed weight, some moisture-related bias cannot be excluded. While this may add random noise to the DMI ground truth data from trial 2, it is unlikely to explain the consistent relationships observed between PAC emissions and intake across diets, but it cannot be discounted that model performances would be higher without this additional source of error.

The strong performance across prediction sets likely reflects both the biological contrasts in DMI between breeds and diets (Figure 1) and the ability of nonlinear algorithms to capture curvilinear relationships, particularly between DMI and CO_2_ production. As in other recent studies, linear models performed less well, while random forest models provided the best balance of accuracy, stability, and interpretability. All algorithms were tuned within the training data to avoid information leakage (having data from the same ewe in both the training and the test data). The random forest approach was therefore retained for further analyses as it performed with the best balance of predictive performance across test sets. We specifically examined the effect of progressively expanding the predictor set. The baseline model (Pred_DMI1) used only PAC-derived traits (CH_4_, CO_2_, O_2_), which are routinely measured at large scale in Norway using custom truck-mounted PAC chambers (Jakobsen et al 2023). Adding BW (Pred_DMI2) improved performance slightly; this trait can be recorded before PAC visits and is already collected periodically on many farms. Including ET (Pred_DMI3) yielded the highest training R^2^ (0.98) and lowest training MAPE (4.3%), although the gain in independent test accuracy was marginal. ET in our study was derived from individual feeding bin data but can also be obtained in grazing sheep via GPS-enabled collar sensors (Versluijs et al 2023).

A substantial gap between training and test performance was observed across all modeling approaches and predictor sets. For example, linear methods such as Lasso and PLSR showed reductions in R^2^ from approximately 0.84 in training to 0.72–0.74 in independent test sets, while more flexible models such as random forest showed a similar decline despite higher training performance (Tables S1–S3). This consistent pattern indicates that the discrepancy is not primarily due to model-specific overfitting but rather reflects the structure of the data and the validation design. In particular, strong contrasts in DMI between breeds and dietary treatments contribute substantially to model generalizability and the variance explained in the training data, inflating apparent predictive performance across all models. In contrast, the identity-blocked test sets, which exclude records from the same ewe, require models to generalize to new individuals and therefore represent a more stringent and realistic evaluation. The marked reduction in performance within breed (eg NWS) further highlights that much of the predictive signal arises from between-group differences rather than within-breed variation.

The modest gains from adding BW and ET are consistent with multi-country findings that PAC traits alone explain most of the variation in feed intake, with BW and feeding behavior contributing incremental but smaller improvements (Graverand et al 2025). Similar patterns were observed by Amarilho-Silveira et al (2025), where CH_4_ and CO_2_ emissions were among the most influential predictors. This consistency across studies supports the biological basis of PAC emissions as strong proxies for energy turnover and feed intake.

Whilst our strategy of separating ewes by identity into training and test sets avoided data leakage from repeated measures, yielding more conservative but realistic performance estimates. It is worth stating that, as with any machine learning approach, validation on truly independent animals is essential to avoid overestimating model utility. Furthermore, although we had 1,830 observations, these were on only 51 unique ewes and fall short of capturing the variation which would, for example, be found in 1,830 observations on 1,830 unique ewes.

### Ranking of sheep for DMI across predictor variables

Accurate ranking of animals for DMI is essential for breeding programs that aim to improve feed efficiency. The breeding value accuracy of any predicted phenotype depends on both measurement precision and its additive genetic correlation (r_G_) with the target trait (Mrode 2003). Gold-standard DMI measurements are costly and logistically prohibitive for large-scale recording in sheep but may be partially replaced by scalable proxy traits if the genetic correlation with true intake exceeds approximately 0.8 (Robertson 1959). Emission traits from PAC, particularly CH_4_ and CO_2_, are directly linked to feed intake through metabolic processes and are already recorded on thousands of sheep in Norway, making them promising proxies for genetic improvement (Jakobsen et al 2023).

At this stage, direct estimation of r_G_ between predicted and measured DMI would require phenotyping thousands of related animals with both traits (Visscher 1998; Bijma and Bastiaansen 2014). In early evaluations, repeatability estimates provide the upper bound for heritability, and individual-level correlations (r_I_) between predicted and measured phenotypes can serve as practical proxies for r_G_ (Falconer and Mackay 1996; Wolak et al 2012). This approach has been applied successfully in ruminant feed-efficiency studies, where individual level correlations between predicted and measured traits were used to approximate genetic associations in advance of large-scale pedigree or genomic data collection (Zetouni et al 2018; Difford et al 2019; Jonker et al 2021). We therefore used r_I_ as an early-stage indicator of the genetic association between measured and predicted DMI, acknowledging that it captures both additive genetic and permanent environmental effects. In the absence of pedigree or genomic data and suitably large datasets of related individuals, r_I_ provides an upper-bound estimate of the potential additive relationship.

This distinction between different correlation coefficients and the inflating effects of between-group differences from fixed effects with the deflating (attenuating) effects of residual error in the presence of repeated measurements is well exemplified by the relationships between O_2_ and DMI. The phenotypic correlation when unadjusted for the effects of breeds and diets in the presence of repeated measurements is very strong (r = −0.80) (Figure 1). After accounting for fixed effects (breed, diet, and trial) but ignoring repeated measurements, this association was substantially reduced (R_P_ = −0.40; Table 4), indicating that part of the observed relationship is driven by between-group differences from the fixed effects rather than within-group variation. The R_I_ after taking into account the fixed effects and repeated measures per individual is -0.62. Thus, the type of correlation used, model assumptions, and the intended purpose can vary greatly and must be interpreted with care. This is particularly important when datasets are selected to increase the between-group variation for training more generalizable models but the intended purpose of the predicted phenotype is to rank animals within groups.

Predicted DMI traits (Pred_DMI1–3) showed strong individual-level correlations (r_I_ = 0.79–0.81) with measured DMI across breeds and diets (Table 4). Breed, diet, and trial were included as fixed effects in the mixed model used to estimate these correlations, ensuring that r_I_ reflects within-breed and within-diet agreement rather than aggregate differences among groups. Despite phenotypic R^2^ increasing slightly with the inclusion of BW and ET, r_I_ remained almost unchanged. This indicates that the apparent gains in overall prediction accuracy mainly reflected fixed between-group effects rather than improved within-animal ranking potential.

Statistically, this pattern is expected when models capture strong environmental contrasts. Increasing contrasts between breeds or diets can inflate overall model R^2^ while leaving individual-level ranking unchanged. Conversely, the presence of repeated measures per ewe introduces two opposing effects: if repeatability is high, leakage of the same individual across folds would inflate performance, whereas if repeatability is below one, within-animal variation introduces noise that can attenuate observed correlations (Adolph and Hardin 2018). By blocking on ewe identity, our validation strategy avoided leakage, and by estimating r_I_ in mixed models that accounted for fixed effects, we mitigated both sources of bias.

The repeatability models indicated that breed and diet nested within trial explained large proportions of phenotypic variance in Pred_DMI1–3, while approximately half of the remaining variance was attributable to consistent individual differences. The similarity in r_I_ values across predictor sets supports that PAC-only predictions already capture most of the biologically meaningful variation in DMI. In contexts where the objective is to rank animals phenotypically for intake, simpler models using only PAC traits may therefore be as effective as more complex multi-predictor models. Future work should estimate genetic (co)variances and r_G_ between measured and predicted DMI in larger, pedigreed populations to quantify the breeding relevance of these predicted traits and to explore additional proxy indicators for feed intake and efficiency.

### Future perspectives

The present study focused on gestating ewes housed indoors under controlled feeding conditions. As such, the study evaluates within-population predictive utility within the breeds and environmental conditions tested. Future work should evaluate whether the observed prediction relationships hold across physiological states such as lactation and growth, where energy balance, metabolism, and feeding behavior differ markedly from those of gestating animals. Extending analyses to growing lambs and lactating ewes will clarify whether the same emission–intake relationships persist across life stages. Estimating phenotypic and genetic correlations between juvenile and adult emission-intake traits will also help determine whether these traits reflect shared biological mechanisms.

Although all data were collected at a single research facility, the experimental design intentionally contrasted two breeds and two silage qualities, introducing biological and management diversity within one site. Future studies should validate these findings under different management systems and environments, including independent farms and pasture-based contexts and Norwegian outfield in mountains, forests, and moors. Linking indoor and pasture PAC measurements could enable development of unified emission–intake indicators for both genetic selection and nutritional management. Across the algorithms tested, random forest achieved the highest predictive performance in the identity-blocked analyses, providing robust results across predictor sets and feeding regimes. Nevertheless, algorithm rankings were derived from a single blocked data partition. Future work will incorporate repeated ewe-identity–blocked resampling and paired statistical comparisons to evaluate model ranking stability and confirm whether the observed random forest advantage persists under replicated analyses.

Finally, additive genetic (co)variances and breeding value accuracies were not estimated in the present study. Quantifying the additive genetic component of predicted DMI traits is a critical next step. This can be achieved through animal models incorporating pedigree or genomic information to estimate genetic correlations (r_G_) and breeding value accuracies relative to directly measured DMI. Such analyses will determine whether PAC-based predicted DMI can serve as a reliable proxy for genetic selection, complementing or potentially replacing direct intake measurements in breeding programs.

## Conclusions

Greenhouse gas emissions measured with PAC accurately predicted DMI across two diets and two breeds in gestating ewes housed indoors at a single research facility. Machine learning models using PAC-derived CH_4_, CO_2_, and O_2_ measurements were able to predict measured DMI with moderate to high accuracy under identity-blocked validation, indicating that these gas traits capture biologically meaningful signals related to feed intake. These results show that PAC-based traits can serve as low-invasive, scalable proxies for feed intake under controlled conditions in gestating ewes. Broader validation across physiological states (lactation, growth) and production environments (including pasture) is required to confirm the generality of these relationships. Pending estimation of additive genetic correlations and breeding value accuracies, PAC-derived predicted DMI could support large-scale genetic evaluations and on-farm benchmarking where direct intake measurements are impractical.

## Supplementary Material

skag158_Supplementary_Data

## Abbreviations

ANN: artificial neural network
BW: body weight
DMI: dry matter intake
EBV: estimated breeding value
ET: eating time
GHG: greenhouse gas
KNN: k-nearest neighbors
MAPE: mean absolute percentage error
NWS: Norwegian White Sheep
ONS: Old Norwegian Spael
PAC: portable accumulation chamber
PLSR: partial least squares regression
RF: random forest
RFID: radio-frequency identification
rG: genetic correlation
rI: individual-level correlation
RMSE: root mean square error
SVM: support vector machine
